# Risk Factors of Multidrug-Resistant Bacteria in Lower Respiratory Tract Infections: A Systematic Review and Meta-Analysis

**DOI:** 10.1155/2020/7268519

**Published:** 2020-06-30

**Authors:** Gang Chen, Kailiang Xu, Fangyuan Sun, Yuxia Sun, Ziyuan Kong, Bangjiang Fang

**Affiliations:** ^1^Department of Intensive Care Medicine, Seventh People's Hospital of Shanghai University of Traditional Chinese Medicine, Shanghai 200137, China; ^2^Department of Emergency, LongHua Hospital of Shanghai University of Traditional Chinese Medicine, Shanghai 200032, China

## Abstract

**Background:**

Multidrug-resistant (MDR) bacteria are the main cause of lower respiratory tract infections (LRTIs) with high mortality. The purpose of this study is to identify the risk factors associated with MDR by performing a systematic review and meta-analysis.

**Methods:**

PubMed, EMBASE (via Ovid), and Cochrane Library were systematically searched for studies on the risk factors for MDR bacteria in LRTIs as of November 30, 2019. Literature screening, data abstraction, and quality assessment of the eligible studies were performed independently by two researchers.

**Results:**

A total of 3,607 articles were retrieved, of which 21 articles representing 20 cohort studies published in English were included after title/abstract and full-text screening. Among the 21 articles involving 7,650 patients and 1,360 MDR organisms, ten reported the risk factors for MDR Gram-positive bacteria (GPB) and Gram-negative bacteria (GNB), ten for MDR GNB, and one for MDR GPB. The meta-analysis results suggested that prior antibiotic treatment, inappropriate antibiotic therapy, chronic lung disease, chronic liver disease and cerebral disease, prior MDR and PA infection/colonization, recent hospitalization, longer hospitalization stay, endotracheal tracheostomy and mechanical ventilation, tube feeding, nursing home residence, and higher disease severity score were independent risk factors for MDR bacteria.

**Conclusions:**

This review identified fourteen clinical factors that might increase the risk of MDR bacteria in patients with LRTIs. Clinicians could take into account these factors when selecting antibiotics for patients and determine whether coverage for MDR bacteria is required. More well-designed studies are needed to confirm the various risk factors for MDR bacteria in the future.

## 1. Introduction

LRTIs are a leading cause of morbidity and mortality around the world. According to the Global Burden of Diseases, Injuries, and Risk Factors (GBD) Study 2017, nearly 2.56 million deaths resulted from LRTIs in 2017, making LRTI the fifth leading cause of mortality for all ages [1]. Besides, more than 50% of LRTI deaths in 2016 were attributable to bacterial aetiologies [2]. Antibiotics are commonly prescribed for antibiotic therapy [3], and antimicrobial resistance is becoming more widely recognized as a leading global health threat [2]. The prevalence of MDR pathogens particularly with carbapenem-resistant *Klebsiella pneumoniae* (CRKP) has sharply increased in recent years, posing significant challenges on anti-infection, and the mortality of patients with MDR bacterial infections was significantly higher than that of patients with non-MDR bacterial infections [4–7]. World Health Organization surveillance reports indicate that the number of patients with LRTIs caused by antibiotic-resistant bacteria continues to increase and such a disease is widespread [8].

Previous studies have found that many risk factors might be associated with the development of MDR bacteria, including prior antibiotic use [9–15], recent hospitalization [15, 16], nursing home residence [10, 11, 15], previous colonization/infection with MDR pathogens [16, 17], ICU stay >7 days [9], APACHE II score [18], chronic pulmonary disease [17, 18], cardiac disease [16, 18], and tracheostomy/mechanical ventilation [19]. The Clinical Practice Guidelines by the Infectious Diseases Society of America and the American Thoracic Society 2016 (IDSA/ATS guidelines 2016) listed the risk factors for MDR pathogens in hospital-acquired pneumonia (HAP) and ventilator-associated pneumonia (VAP) [20]. Due to the emergence of new studies in recent years [14, 17], no review has systematically summarized the risk factors for MDR bacterial infection in patients with LRTIs, so we conducted this systematic review and meta-analysis to identify the risk factors associated with MDR bacteria in patients with LRTIs and to provide evidence for clinical practice.

## 2. Materials and Methods

We conducted the study following the Preferred Reporting Items for Systematic Review and Meta-Analyses (PRISMA) guidelines [21]. There was no requirement for ethical approval because we analyzed scientific literature already in the public domain.

### 2.1. Search Strategy

PubMed, EMBASE (via Ovid), and Cochrane Library were searched systematically for studies on the risk factors for MDR bacteria in LRTIs up to November 30, 2019. The free text words such as “Gram-Negative Bacteria,” “Acinetobacter baumannii,” “Pseudomonas aeruginosa,” “Escherichia coli,” “Klebsiella pneumoniae,” “Methicillin-Resistant Staphylococcus aureus,” “MRSA,” “Enterobacteriaceae,” “Carbapenem-Resistant Enterobacteriaceae,” “Multiple Drug Resistance,” “Respiratory Tract Infections,” “Pneumonia,” “Hospital-acquired pneumonia,” “Ventilator-associated pneumonia,” “Community-acquired pneumonia (CAP),” and “Bronchopneumonia” and Medical Subject Headings (MeSH) were combined with the Boolean operators “AND” or “OR.” We listed the detailed retrieval strategies in Tables S1–S3 in the Supplementary Materials. Additionally, we checked through the reference lists of relevant studies to see if these references include reports of other studies that might be eligible for the review.

### 2.2. Study Selection

We had access to all published articles that evaluated the risk factors for MDR bacteria in LRTIs and included prospective or retrospective cohorts that included adult patients with LRTIs and were published in English. We excluded studies if they were case reports, case series, animal studies, or review; if they included nonrespiratory tract infection patients or pediatric patients; if they reported MDR organisms less than ten cases; if they merely reported the results of unadjusted analysis; and if the full texts of them were unavailable. For articles that covered the same population as other articles, if the articles provided new information, we considered them were qualified, and if not, we chose the article with better homogeneity when it was synthesized with other studies.

The literature selection was performed independently by two researchers (G. C and KL. X), and any disagreements were resolved by consensus. We accepted MDR organisms as defined by individual studies, even if the definitions were inconsistent across studies. One definition of MDR is the development of resistance to more than three antibiotic classes known to be active against these pathogens [22] (Definition A), and the other considered methicillin-resistant *Staphylococcus aureus* (MRSA), extended-spectrum beta-lactamases (ESBLs), CRKP and *Escherichia coli* (Eco), *Pseudomonas aeruginosa* (PA), *Acinetobacter baumannii* (AB), and *Stenotrophomonas maltophilia* (SMA) as MDR pathogens according to the IDSA/ATS Guidelines 2005 [23] (Definition B). Besides, some studies did not elaborate on the definition of MDR bacteria (No definition). In the review, the hospital setting comprised all types of units, including intensive care units, emergency room/casualty, or other wards.

### 2.3. Data Abstraction

Two researchers (G.C and KL.X) independently extracted the following information: author, year of publication, countries, type of study, setting, sample sizes, the definition of MDR, age, and all reported risk factors. If no consensus can be reached on the disagreement, another reviewer would participate in the decision-making. We used standardized data extraction sheets made by Microsoft Excel 2019 for data extraction.

### 2.4. Risk of Bias Assessment of Eligible Studies

We conducted the risk of bias assessment of eligible studies based on Quality in Prognosis Studies (QUIPS) tool [24]. The risk of bias assessment covered the following six domains: study participation, study attrition, prognostic factor measurement, outcome measurement, study confounding, and statistical analysis and reporting. Considering all relevant issues, each of these six domains can be rated as the one with high, medium, or low potential deviation risk. For the overall rating, we followed the approach from Foroutan et al. [25] and classified studies with five or six low-risk domains as at overall low risk of bias, studies with two or more high-risk domains as at overall high risk of bias, and remaining studies as at overall moderate risk of bias.

### 2.5. Statistical Analysis

We calculated the pooled odds ratio (OR) with a 95% confidence interval (CI). The *Q* statistic (significant when *P* < 0.10) was employed to explore the heterogeneity across studies, and then the *I*^2^ statistic was used to quantify the extent of heterogeneity. We considered that *I*^2^ > 50% represented substantial inconsistency or significant statistical heterogeneity, and data synthesis was performed in the DerSimonian and Laird (DL) random-effects model. Subsequently, subgroup analyses were conducted according to diagnosis (pneumonia, CAP, HAP, VAP, and other LRTIs), kinds of organisms (CPB, GNB, and CPB and GNB), and definitions of MDR (Definition A, Definition B, and No definition) to explore the sources of heterogeneity. Moreover, fixed-effects model and Hartung–Knapp–Sidik–Jonkman (HKSJ) random-effects model were used to perform sensitivity analysis in order to verify the robustness of the meta-analysis results. The potential publication bias of eligible studies was identified using Begg's test and Egger's test for risk factors reported in nine or more studies. We considered a *P* value of less than 0.05 to be statistically significant. The trim and fill method was applied to adjust the results of the pooled analysis in the case of publication bias. All statistical tests were two-sided. Statistical software Stata version 14.0 (StataCorp, College Station, TX, 2014) and R version 4.0.0 were employed in the review.

## 3. Results

**Figure 1:**
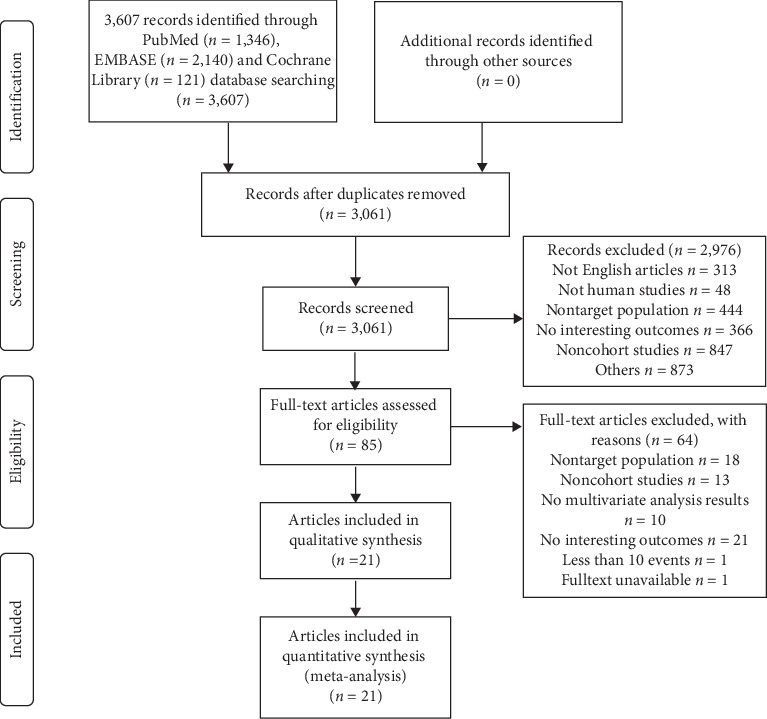
PRISMA 2009 flow diagram in literature screening; nontarget population refers to nonrespiratory tract infection patients or pediatric patients; no interesting outcomes refer to no adjusted analysis results for risk factors were reported in eligible studies.

A total of 3, 607 articles were retrieved, and 21 articles [14–17, 19, 26–41] representing 20 cohort studies published in English between 2006 and 2019 were included after title/abstract and full-text screening. The PRISMA 2009 flow diagram in literature screening can be referred to Figure 1. Studies excluded in the full-text screening process are listed in Table S2 in the Supplementary Materials.

Among the 21 eligible articles, nine were prospective studies [14, 16, 17, 19, 32, 37, 39–41] and twelve were retrospective studies [26–31, 33–36, 38]. Three articles [16, 17, 31] were conducted at multiple centers. Except for an international multicenter study [16, 17], all other studies [14, 15, 19, 26–41] were confined to one country. The majority of the populations included were patients with pneumonia [14–17, 26–29, 31–41]. The mean age of the study subjects ranged from 42.6 years to 74.9 years, and the males accounted for 44.5% to 89.7% of all the study subjects. We summarized the baseline characteristics of the included studies in Table 1. The 21 eligible articles involved 7,650 patients and 1,360 MDR organisms, of which ten reported the risk factors for MDR GPB and GNB [15, 19, 26, 28, 31, 33, 34, 38, 39, 41], ten for MDR GNB [14, 16, 17, 27, 29, 30, 32, 35, 36, 40], and one for MDR GPB [37].

The risk of bias of the included articles is listed in Table 2. The overall risk of bias of 15 articles [14–17, 19, 27, 29–31, 33, 35, 36, 38, 39, 41] is rated as low, four [26, 34, 37, 40] as moderate, and two [28, 32] as high, indicating that most of the included studies had intermediate or higher quality.

### 3.1. Meta-Analysis Results

In the meta-analysis, the risk factors significantly associated with the acquisition of MDR bacteria are described in the Results section. Additionally, other risk factors that are statistically significantly associated with the acquisition of MDR bacteria but are not suitable for meta-analysis are listed in Table S3 in the Supplementary Materials.

### 3.2. Antibiotic Treatment

**Figure 2:**
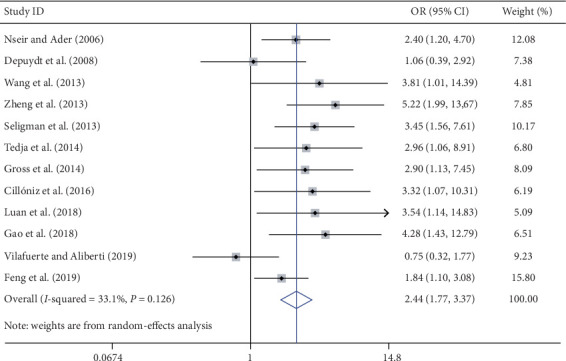
Forest plot of the meta-analysis regarding the MDR bacterial infection due to prior antibiotic treatment in the random-effects model. OR: odds ratio; CI: confidence interval.

Prior antibiotic treatment is the most frequently reported risk factor that is correlated with the acquisition of MDR bacteria [14–16, 19, 26, 28, 30, 34, 36–38, 41]. Six of the included studies [14, 19, 30, 36, 38, 41] defined prior antibiotic treatment as the antibiotic use within 30 days before admission, while another two studies [15, 26] as 90 days before diagnosis and one study [16] as 12 months before diagnosis. The remaining three studies [28, 34, 37] did not specify the period between the diagnosis and the last antibiotic treatment. In the meta-analysis, twelve studies [14–16, 19, 26, 28, 30, 34, 36–38, 41] indicated that prior antibiotic treatment had a statistically significant association with the acquisition of MDR bacteria (OR: 2.35; 95% CI: 1.92 to 3.18; *I*^2^ = 20.8%) in the random-effects model (Figure 2). Besides, two studies [27, 29] demonstrated that inappropriate antibiotic therapy was also associated with an increased risk of acquisition (OR: 14.99; 95% CI: 8.56 to 26.26.12; *I*^2^ = 41.1%) using the random-effects model.

### 3.3. Comorbidities

In patients, chronic lung disease [16, 17, 26, 36, 38] including chronic obstructive pulmonary disease [17, 26, 38] and chronic liver disease [16, 32], cerebral disease [33, 36, 40] including encephalopathy grades II–IV [40], and cerebrovascular events [33, 36] could increase the risk of the development of MDR bacteria, while cardiac disease [33, 36, 40] and kidney disease requiring renal replacement therapy [15, 38, 41] did not add the risk of MDR bacterial infection. The results of the meta-analysis are listed in Table 3.

### 3.4. Prior Infection/Colonization

Three studies [15, 16, 33] demonstrated that patients with prior MDR infection/colonization in the previous 12 months (OR: 3.80; 95% CI: 1.53 to 9.41; *I*^2^ = 60.8%) had a significantly increased risk of being infected with MDR bacteria in the random-effects model, so did the patients with prior PA infection/colonization (OR: 10.29; 95% CI: 5.03 to 21.07; *I*^2^ = 0.0%) reported in two studies [15, 17].

### 3.5. Hospitalization

For recent hospitalization, two of the included studies [15, 31] defined it as hospitalization within the last 3 months, while one study [16] as 12 months and one study [26] did not specify the meaning of “recent”.

In the random-effects model, recent hospitalization [15, 16, 26, 31] (OR: 2.47; 95% CI: 1.47 to 4.15: *I*^2^ = 10.6%, four studies) or healthcare exposure prior to admission [15, 33, 34] (OR: 3.10; 95% CI: 1.94 to 4.97; *I*^2^ = 0.0%, three studies) was identified with increased odds of MDR bacterial infection. Moreover, the longer the hospitalization stay, the greater the risk of infection with MDR bacteria (OR: 1.03; 95% CI: 1.01 to 1.06; *I*^2^ = 76.3%, four studies) [15, 34, 35, 41].

### 3.6. Hospital Interventions

Previous or present endotracheal intubation increased the risk of MDR bacteria obviously (OR: 6.56; 95% CI: 1.03 to 41.94; *I*^2^ = 91.3%, three studies) [19, 26, 40], which was also applicable to patients treated with mechanical ventilation (OR: 7.97; 95% CI: 2.41 to 26.33; *I*^2^ = 65.1%, three studies) [17, 33, 36]. In patients requiring enteral nutritional support, tube feeding (OR: 2.95; 95% CI: 1.12 to 7.80; *I*^2^ = 0.0%, two studies) [16, 31] was also a factor that increased the risk of MDR bacteria.

### 3.7. Others

Furthermore, the higher the disease severity scores, the higher the risk of the infection with MDR bacteria (OR: 2.29; 95% CI: 1.41 to 3.74; *I*^2^ = 11.2%, two studies) [35, 36], and neither HAP nor VAP was a risk factor for the increased MDR bacterial infection (OR: 1.79; 95% CI: 0.64 to 5.02; *I*^2^ = 29.1%, three studies) [15, 29, 31] in the random-effects model.

### 3.8. Publication Bias

For studies included in the meta-analysis which assessed prior antibiotic treatment [14–16, 19, 26, 28, 30, 34, 36–38, 41] as a risk factor, the quantitative evaluation of publication bias by Begg's (*P*=0.115) and Egger's (*P*=0.125) tests indicated that the publication bias was not statistically significant, thus suggesting that there was no publication bias for the studies that reported prior antibiotic treatment as a risk factor.

We subdivided studies reporting prior antibiotic treatment into different groups, from which we obtained similar results to the overall results when the eligible studies were divided by kinds of organisms or definitions of MDR. Meanwhile, the subgroup analysis divided by diagnosis showed that prior antibiotic treatment was a risk factor for the acquisition of MDR bacteria in patients with HAP [26, 37, 38], VAP [34, 41], pneumonia [15, 36], bronchiectasis [30], and AECOPD [42] except for CAP [14, 16, 28]. In the CAP subgroup [14, 16, 28], the pooled analysis results suggested that prior antibiotic treatment was significantly associated with MDR bacterial infection after we ruled out the adjusted OR value of the study of Villafuerte et al. [16], which defined prior antibiotic treatment as antibiotics use during the last 12 months, far longer than the time frames reported in other studies. The results of the subgroup analysis are presented in Table 4.

### 3.9. Sensitivity Analysis

Compared with the use of the random-effects model (DL method), when we performed data synthesis using the fixed-effects model, the pooled adjusted ORs for all risk factors did not change significantly. Nine risk factors obtained no significant result in the HKSJ method for meta-analysis, while results were statistically significant using the DL method. The results of sensitivity analysis using the fixed-effects model and HKSJ random-effects model are presented in Tables S6 and S7.

## 4. Discussion

LRTIs are highly prevalent and variable and confer considerable morbidity and mortality [43]. The increasing rates of MDR bacteria are a worldwide public health problem. This review indicates that such risk factors as prior antibiotic treatment, inappropriate antibiotic therapy, chronic lung disease, chronic liver disease, cerebral disease, prior MDR infection/colonization, recent hospitalization, longer duration of hospitalization, previous or present endotracheal intubation or mechanical ventilation, tube feeding, and higher disease severity scores had a statistically significant association with the acquisition of MDR bacterial infection.

For antibiotic treatment, the present study found that prior antibiotic treatment was a significant risk factor for MDR bacterial infection in LRTIs. This finding is similar to that of the previous studies, which identified the risk factors for MDR PA infection in hospitalized patients [44] and MDR GNB infection in intensive care units [45]. The above two systematic reviews could not specify the definitions of previous antibiotic use, nor can they conclude an exact cutoff point for the time frame. Even if the eligible studies had inconsistent definitions of prior antibiotic treatment, we suggest defining it as the use of antibiotics within 90 days in clinical practice. The IDSA/ATS Guidelines 2016 [20] also suggested that the prior intravenous antibiotic use within 90 days was an important factor for HAP and VAP. Studies [46–48] have consistently reported that inappropriate antibiotic therapy, such as overuse or underuse of empirical antibiotics, could result in an increase in drug-resistant bacteria and generate new disease burdens [49]. The carbapenem-resistant Enterobacteriaceae (CRE), whose continuous emergence over the past decade has caused global attention, is significantly related to the increasing use of carbapenems [47, 50]. Two meta-analyses [51, 52] proved that exposure to carbapenems could increase the risk of CRKP by three to four times. Owing to the high mortality of patients with CRKP and fewer alternative treatment options, experts have proposed to limit the excess use of carbapenems [53].

The IDSA/ATS Guidelines 2016 [20] proposed that five or more days of hospitalization prior to the occurrence of VAP was considered a factor that increased the risk of MDR bacterial infection. This review confirms that recent hospitalization and prolonged hospital stays can increase the risk of MDR bacteria. A meta-analysis [51] suggested that prior hospitalization (within the previous 6 months) was a predictor for CRKP infection, and the present review indicated the appropriate cutoff value of the time frame for recent hospitalization was 90 days. Furthermore, we did agree that prolonged hospital stay could increase the risk of MDR bacterial infections [51].

Patients with comorbidities, such as chronic respiratory disease (COPD, asthma, and bronchiectasis), chronic liver disease, and cerebral disease are associated with the development of MDR bacteria, as they are particularly susceptible to bacterial infections and usually require repeated hospitalizations, antibiotic treatment, and invasive procedures [42, 54]. The same is true for more severe patients who have higher disease severity scores. Besides, studies have shown that the colonization of GNB, especially PA, was common in patients with chronic respiratory disease. It is prone to recurrent infection [55–57], followed by frequent antibiotic exposure, which positively selects for MDR bacteria. The IDSA/ATS Guidelines 2016 [20] and Zhu et al. [51] pointed out that the patients with renal dysfunction or dialysis were at increased risk of infection with MDR bacteria, which had not been confirmed in this study.

Consistent with previous meta-analysis [51, 52], we found that intubation/ventilation (within the previous 6 months) and prior MDR bacterial infection/colonization (within the previous 6 months) were significantly associated with the increase of MDR bacteria.

The sensitivity analysis suggested that the results of the HKSJ method did not fully agree with those of the DL method. Nine significant risk factors including inappropriate antibiotic therapy, chronic liver disease, cerebral disease, prior MDR infection, prior PA infection, endotracheal intubation, mechanical ventilation, tube feeding, and disease severity scores did not obtain significant results when using the HKSJ method, although the studies included in the meta-analysis were statistically significant with effects pointing into the same direction. This might be because the risk factors only reported by very few (i.e., 2 or 3) studies, and the HKSJ method had very low power and leads to a statistically not significant pooled effect estimate [58, 59]. The meta-analysis results of the above nine risk factors should be regarded with caution.

To the best of our knowledge, this review is the first one that conducts a meta-analysis by focusing on risk factors for MDR bacteria in LRTIs, but there are also some limitations. Firstly, due to the limited number of included studies, subgroup analyses based on diagnosis, kinds of organisms, or definitions of MDR bacteria were not conducted for most risk factors. Thus, it might limit the generalizability of the results. Secondly, the different definitions of MDR bacteria used in the original literature may introduce deviations in the results, even though most studies had the same definition that the MDR bacteria were not sensitive to at least one agent in three or more antimicrobial categories. Thirdly, some factors including previous antibiotic treatment, recent hospitalization, and previous tracheostomy were not defined consistently across studies and even were not defined clearly in some studies, leading to the reduction in the precision of the results. Fourthly, limited evidence exists to inform which method performs best for a random-effects meta-analysis, especially when studies are few in number (<5). Therefore, we applied the commonly used random-effects model (DL method) for our primary analysis. The DL method might generate too many statistically significant results when the number of studies is small and there is moderate or substantial heterogeneity [60]. To ensure the robustness of the meta-analysis results, HKSJ random-effects model was used for sensitivity analysis.

## 5. Conclusions

This meta-analysis indicates that prior antibiotic treatment in the past 90 days, inappropriate antibiotic therapy, chronic lung disease, chronic liver disease, cerebral disease, prior MDR infection/colonization in the past 12 months, hospitalization in the past 90 days, longer hospitalization stay, previous endotracheal intubation or mechanical ventilation in the past 6 months, tube feeding, and higher the disease severity scores were risk factors for the acquisition of MDR bacteria. Clinicians could take into account these factors when selecting antibiotics for patients and determine whether coverage for MDR is required in clinical practice. More well-designed studies are needed to confirm the various risk factors for MDR bacteria in the future.

## Figures and Tables

**Table 1:** The baseline characteristics of included studies.

Study ID	Year	Study type	Countries	No. of centers	Setting	Population	No. of patients	No. of MDROs	MDR strains	Age (years)^a^	Male (%)
Villafuerte and Aliberti [16]	2019	Prospective	54 countries	222	Internal and emergency medicine, infectious diseases, critical care and pulmonary medicine	CAP	3193	38	EB	68.0 (54.0, 80.0)	58.8
Restrepo et al. [17]	2017	Prospective	54 countries	222	Internal and emergency medicine, infectious diseases, critical care and pulmonary medicine	CAP	133	33	PA	64.4 (52.5, 78.5)	59.4
Feng et al. [26]	2019	Retrospective	China	Single	Pulmonary and critical care medicine, surgical ICU	HAP	313	193	EB, SA	NA	72.5
						VAP	106	78	EB, SA	NA	67.0
Zhou et al. [27]	2018	Retrospective	China	Single	Institute of Respiratory Diseases, Division of Respiratory Diseases of Department of Internal Medicine, Department of Pediatrics	HAP	157	69	PA	57.8 ± 17.8	71.3
Luan et al. [28]	2018	Retrospective	China	Single	Department of Infectious Diseases	CAP	176	29	EB, SA, SP	68.3 ± 4.3	53.4
Lewis et al. [29]	2018	Retrospective	USA	Single	Trauma ICU	VAP	397	135	AB, PA	45.0 (16.0, 85.0)	78.0
Gao et al. [30]	2018	Retrospective	China	Single	Department of Respiratory and Critical Care Medicine, Department of Emergency Medicine	Bronchiectasis	88	34	PA	59.7 ± 18.2	52.3
Song et al. [31]	2017	Retrospective	Korea	3	NA	HDAP	105	24	GPB & GNB	71.0 (61.0, 76.0)	64.8
Fernandez-Barat et al. [32]	2017	Prospective	Spain	Single	Medical and surgical ICUs	ICUAP	64	22	PA	66.0 ± 15.0	73.4
Huang et al. [33]	2016	Retrospective	China	Single	Medical ICU	Pneumonia	263	154	GPB & GNB	72.9 ± 14.1	62.3
Cillóniz et al. [14]	2016	Prospective	Spain	Single	Hospital clinic	CAP	77	22	PA	71.4 ± 14.6	84.4
Tedja et al. [34]	2014	Retrospective	USA	Single	Medical, surgical, cardiovascular, coronary, and neurologic ICU	VAP	107	49	GPB & GNB	62.0 ± 14.0	55.0
Özgür et al. [35]	2014	Retrospective	Turkey	Single	Medical, surgical, adult ICU	VAP	134	34	AB	53.2 ± 21.0	59.0
Gross et al. [15]	2014	Retrospective	USA	Single	Academic medical center	CAP or HCAP	521	20	GPB & GNB	65.0 (52.0, 79.0)	44.5
Wang et al. [37]	2013	Prospective	China	Single	Tertiary teaching hospital	HAP	102	24	MRSA	74.9 ± 12.4	64.7
Zheng et al. [36]	2013	Retrospective	China	Single	Hospital affiliated to a university	Pneumonia	242	97	AB	61.4 ± 9.8	54.9
Seligman et al. [38]	2013	Retrospective	Brazil	Single	Tertiary care teaching hospital	HAP	140	59	GPB & GNB	63.0 ± 14.4	70.0
Hamet et al. [39]	2012	Prospective	France	Single	ICU	VAP	323	90	GPB & GNB	63.4 ± 15.2	66.9
Shi et al. [40]	2010	Prospective	China	Single	Hospital affiliated to a university	Pneumonia	475	57	GNB	42.6 ± 11.3	89.7
Depuydt et al. [41]	2008	Prospective	Belgium	Single	Medical and surgical ICU	VAP	192	52	GPB & GNB	59.4 ± 16.1	71.9
Nseir and Ader [42]	2006	Prospective	France	Single	ICU	AECOPD	788	69	GPB & GNB	66.2 ± 11.9	76.8

^a^Mean ± SD or median (IQR); MDR: multidrug resistance; MDROs: multidrug-resistant organisms; no.: number; ICU: intensive care unit; CAP: community-acquired pneumonia; HAP: hospital-acquired pneumonia; VAP: ventilator-associated pneumonia; HDAP: hemodialysis-associated pneumonia; ICUAP: intensive care unit-acquired pneumonia; HCAP: healthcare-associated pneumonia; AECOPD: acute exacerbation of chronic obstructive pulmonary disease; GNB: Gram-negative bacteria; GPB and GNB: Gram-negative bacteria and Gram-positive bacteria; EB: Enterobacteriaceae; PA: *Pseudomonas aeruginosa*; SA: *Staphylococcus aureus*; SP: *Streptococcus pneumoniae*; MRSA: methicillin-resistant *Staphylococcus aureus*; AB: *Acinetobacter baumannii*.

**Table 2:** Risk of bias assessment of eligible studies based on QUIPS tool.

Study ID	Study participation	Study attrition	Prognostic factor measurement	Outcome measurement	Study confounding	Statistical analysis and reporting	Overall risk of bias
Vilafuerte and Aliberti [16]	Low	Low	Low	Low	Low	Low	Low
Restrepo et al. [17]	Low	Low	Low	Low	Low	Low	Low
Feng et al. [26]	Low	Low	Moderate	Low	Moderate	Low	Moderate
Zhou et al. [27]	Low	Low	Low	Low	Low	Low	Low
Luan et al. [28]	Low	Low	High	High	Low	Low	High
Lewis et al. [29]	Low	Low	Low	Low	Low	Low	Low
Gao et al. [30]	Low	Low	Low	Low	Low	Low	Low
Song et al. [31]	Low	Low	Low	Low	Low	Low	Low
Fernandez-Barat et al. [32]	Low	Low	High	High	Low	Low	High
Huang et al. [33]	Low	Low	Low	Low	Low	Low	Low
Cillóniz et al. [14]	Low	Low	Low	Moderate	Low	Low	Low
Tedja et al. [34]	Low	Low	Moderate	Moderate	Moderate	Low	Moderate
Özgüret al. [35]	Low	Low	Low	Low	Low	Low	Low
Grosset al. [15]	Low	Low	Low	Low	Low	Low	Low
Wang et al. [37]	Moderate	Low	Moderate	Low	Moderate	Low	Moderate
Zheng et al. [36]	Moderate	Low	Low	Low	Low	Low	Low
Seligman et al. [38]	Moderate	Low	Low	Low	Low	Low	Low
Hamet et al. [39]	Low	Low	Low	Low	Low	Low	Low
Shi et al. [40]	Moderate	Low	Low	Low	Moderate	Low	Moderate
Depuydt et al. [41]	Low	Low	Low	Low	Low	Low	Low
Nseir and Ader [42]	Low	Low	Low	Low	Low	Low	Low

**Table 3:** Risk factors of MDR bacteria in terms of comorbidities

Risk factors	No. of included studies	No. of included MDROs	Heterogeneity	Synthesized results^*∗*^
Chronic lung disease [16, 17, 26, 36, 38]	5	420	*I* ^2^ = 0.0%, *P*=0.611	OR: 2.19; 95% CI: 1.51 to 3.19
Chronic liver disease [16, 32]	2	60	*I* ^2^ = 0.0%, *P*=0.403	OR: 3.41; 95% CI: 1.55 to 7.51
Cardiac disease [16, 38]	2	97	*I* ^2^ = 57.9%, *P*=0.123	OR: 0.67; 95% CI: 0.25 to 1.86
Cerebral disease [33, 36, 40]	3	308	*I* ^2^ = 73.2%, *P*=0.024	OR: 2.98; 95% CI: 1.37 to 6.50
Renal replacement therapy [15, 38, 41]	3	131	*I* ^2^ = 0%, *P*=0.559	OR: 0.78; 95% CI: 0.41 to 1.48

MDROs: multidrug-resistant organisms. ^*∗*^Using random-effects model.

**Table 4:** Subgroup analysis of studies reporting prior antibiotic treatment as a risk factor.

Subgroups	No. of included studies	Heterogeneity	Synthesized results^*∗*^
Divided by diagnosis			
CAP [14, 16, 28]	3	*I* ^2^ = 67.1%, *P*=0.048	OR: 1.92; 95% CI: 0.65 to 5.70
HAP [26, 37, 38]	3	*I* ^2^ = 11.8%, *P*=0.322	OR: 2.40; 95% CI: 1.52 to 3.80
VAP [34, 41]	2	*I* ^2^ = 0.0%, *P*=0.169	OR: 2.63; 95% CI: 1.24 to 5.55
Pneumonia [15, 36]	2	*I* ^2^ = 0.0%, *P*=0.170	OR: 3.87; 95% CI: 1.97 to 7.59
Bronchiectasis [30]	1	NA	OR: 4.28; 95% CI: 1.43 to 12.80
AECOPD [42]	1	NA	OR: 2.40; 95% CI: 1.21 to 4.75

Divided by kinds of organisms			
GNB [14, 16, 30, 36]	4	*I* ^2^ = 72.7%, *P*=0.012	OR: 2.65; 95% CI: 1.01 to 6.92
GNB and GPB [16, 26, 28, 34, 38, 41, 42]	7	*I* ^2^ = 0.0%, *P*=0.559	OR: 2.44; 95% CI: 1.81 to 3.30
GPB [37]	1	NA	OR: 3.81; 95% CI: 1.01 to 14.39

Divided by definitions of MDR			
Definition A [14, 16, 26, 30, 34, 36–38, 41]	9	*I* ^2^ = 40.4%, *P*=0.098	OR: 2.57; 95% CI: 1.72 to 3.82
Definition B [15, 42]	2	*I* ^2^ = 0.0%, *P*=0.750	OR: 2.56; 95% CI: 1.47 to 4.45
No definition [28]	1	NA	OR: 3.54; 95% CI: 0.98 to 12.77

^*∗*^Using random-effects model; NA: not applicable; CAP: community-acquired pneumonia; HAP: hospital-acquired pneumonia; AECOPD: acute exacerbation of chronic obstructive pulmonary disease; GNB: Gram-negative bacteria; GPB: Gram-positive bacteria; GNB and GPB: Gram-negative bacteria and Gram-positive bacteria; MDROs: multidrug-resistant organisms; Definition A: MDROs defined as resistance to ≥3 antimicrobial classes known to be active against these pathogens; Definition B: MDROs defined as methicillin-resistant *Staphylococcus aureus*, *Pseudomonas aeruginosa*, extended-spectrum *β*-lactamase-producing and carbapenem-resistant *Klebsiella pneumoniae* and *Escherichia coli*, AB, and *Stenotrophomonas maltophilia*; No definition: no elaboration on the definition of MDROs.

## Supplementary Materials

Supplementary Materials Table S1: search strategies in PubMed. Table S2: search strategies in EMBASE. Table S3: search strategies in Cochrane Library. Table S4: studies excluded in the full-text screening process. Table S5: additional risk factors that had a statistically significant association with the acquisition of MDR bacteria. Table S6: results of sensitivity analysis using the fixed-effects models. Table S7: results of sensitivity analysis using the HKSJ random-effects model.
