# Supplementary material for: Risk Factors of Multidrug-Resistant Bacteria in Lower Respiratory Tract Infections: A Systematic Review and Meta-Analysis
Source: Can J Infect Dis Med Microbiol. 2020 Jun 30;2020:7268519. doi: 10.1155/2020/7268519 (PMC7345606; doi:10.1155/2020/7268519)
Supplement: Supplementary Materials — Table S1: search strategies in PubMed. Table S2: search strategies in EMBASE. Table S3: search strategies in Cochrane Library. Table S4: studies excluded in the full-text screening process. Table S5: additional risk factors that had a statistically significant association with the acquisition of MDR bacteria. Table S6: results of sensitivity analysis using the fixed-effects models. Table S7: results of sensitivity analysis using the HKSJ random-effects model. [file 7268519.f1.docx]

**Table S1 Search strategies in PubMed**

| **Query** | **Items found** |
| --- | --- |
| Search (((((((((((((((((((((((("Gram-Negative Bacteria"[Mesh]) OR Gram-Negative Bacteria[Title/Abstract]) OR gram-negative bacillus[Title/Abstract]) OR gram-negative bacterium[Title/Abstract]) OR gram negative bacteria[Title/Abstract])) OR GNB[Title/Abstract]) OR "Acinetobacter baumannii"[Mesh])) OR Acinetobacter baumannii[Title/Abstract]) OR acinetobacter baumanii[Title/Abstract]) OR "Pseudomonas aeruginosa"[Mesh]) OR Pseudomonas aeruginosa[Title/Abstract]) OR "Escherichia coli"[Mesh]) OR escherichia coli[Title/Abstract]) OR e.coli[Title/Abstract]) OR "Klebsiella pneumoniae"[Mesh]) OR klebsiella pneumoniae[Title/Abstract]) AND Humans[Mesh])) OR (((((((("Staphylococcus aureus"[Mesh]) OR Staphylococcus aureus[Title/Abstract]) OR "Methicillin-Resistant Staphylococcus aureus"[Mesh]) OR ((Methicillin-Resistant Staphylococcus aureus[Title/Abstract]) OR MRSA[Title/Abstract])) OR "Enterobacteriaceae"[Mesh]) OR Enterobacteriaceae[Title/Abstract]) OR "Carbapenem-Resistant Enterobacteriaceae"[Mesh]) OR Carbapenem-Resistant Enterobacteriaceae[Title/Abstract])) AND Humans[Mesh])) AND ((((((("Drug Resistance, Multiple"[Mesh]) OR Multiple Drug Resistance[Title/Abstract]) OR Resistance, Multiple Drug[Title/Abstract]) OR Multidrug Resistance[Title/Abstract]) OR Multi-Drug Resistance[Title/Abstract]) OR Drug Resistance, Multiple[Title/Abstract]) AND Humans[Mesh])) AND Humans[Mesh])) AND ((((((((((((((("Respiratory Tract Infections"[Mesh]) OR Respiratory Tract Infections[Title/Abstract]) OR "Pneumonia"[Mesh]) OR Pneumonia[Title/Abstract]) OR "Bronchopneumonia"[Mesh]) OR "Healthcare-Associated Pneumonia"[Mesh]) OR Healthcare-Associated Pneumonia[Title/Abstract]) OR "Pleuropneumonia"[Mesh]) OR "Pneumonia, Ventilator-Associated"[Mesh]) OR Pneumonia, Ventilator-Associated[Title/Abstract]) OR VAP[Title/Abstract]) OR HAP[Title/Abstract]) OR "Pneumonia, Bacterial"[Mesh]) OR Pneumonia, Bacterial[Title/Abstract])) AND Humans[Mesh]) Filters: Humans | 1346 |

**Table S2 Search strategies in Embase**

|  | Query | Items found |
| --- | --- | --- |
| 1 | exp Gram negative bacterium/ | 783203 |
| 2 | Gram negative bacterium.ab,kw,ti. | 4569 |
| 3 | gram-negative bacillus.ab,kw,ti. | 1139 |
| 4 | Gram-Negative Bacteria.ab,kw,ti. | 37654 |
| 5 | GNB.ab,kw,ti. | 1506 |
| 6 | exp Acinetobacter baumannii/ | 14462 |
| 7 | exp Pseudomonas aeruginosa/ | 93391 |
| 8 | exp Klebsiella pneumoniae/ | 39744 |
| 9 | exp Escherichia coli/ | 350279 |
| 10 | exp Staphylococcus aureus/ or exp methicillin resistant Staphylococcus aureus/ | 162348 |
| 11 | exp carbapenemase producing Enterobacteriaceae/ or exp extended spectrum beta lactamase producing Enterobacteriaceae/ or exp carbapenem-resistant Enterobacteriaceae/ or exp Enterobacteriaceae/ or exp multidrug resistant Enterobacteriaceae/ | 470507 |
| 12 | Acinetobacter baumannii.ab,kw,ti. | 10469 |
| 13 | Pseudomonas aeruginosa.ab,kw,ti. | 69839 |
| 14 | Klebsiella pneumoniae.ab,kw,ti. | 25315 |
| 15 | Escherichia coli.ab,kw,ti. | 268887 |
| 16 | e coli.ab,kw,ti. | 150745 |
| 17 | Staphylococcus aureus.ab,kw,ti. | 121347 |
| 18 | methicillin resistant Staphylococcus aureus.ab,kw,ti. | 26633 |
| 19 | carbapenemase producing Enterobacteriaceae.ab,kw,ti. | 1023 |
| 20 | extended spectrum beta lactamase producing Enterobacteriaceae.ab,kw,ti. | 793 |
| 21 | carbapenem-resistant Enterobacteriaceae.ab,kw,ti. | 1685 |
| 22 | Enterobacteriaceae.ab,kw,ti. | 21550 |
| 23 | multidrug resistant Enterobacteriaceae.ab,kw,ti. | 251 |
| 24 | 12 or 13 or 14 or 15 or 16 or 17 or 18 or 19 or 20 or 21 or 22 or 23 | 494751 |
| 25 | 1 or 2 or 3 or 4 or 5 or 6 or 7 or 8 or 9 or 10 or 11 or 24 | 970239 |
| 26 | exp multidrug resistance/ | 44777 |
| 27 | Multiple Drug Resistance.ab,kw,ti. | 2694 |
| 28 | 26 or 27 | 45990 |
| 29 | 25 and 28 | 16280 |
| 30 | exp respiratory tract infection/ | 377940 |
| 31 | exp community acquired pneumonia/ or exp ventilator associated pneumonia/ or exp pneumonia/ or exp bacterial pneumonia/ or exp health care associated pneumonia/ | 281879 |
| 32 | respiratory tract infection.ab,kw,ti. | 14589 |
| 33 | pneumonia.ab,kw,ti. | 161699 |
| 34 | CAP.ab,kw,ti. | 56213 |
| 35 | HAP.ab,kw,ti. | 6503 |
| 36 | VAP.ab,kw,ti. | 6730 |
| 37 | 30 or 31 or 32 or 33 or 34 or 35 or 36 | 638644 |
| 38 | 29 and 37 | 2140 |

**Table S3 Search strategies in Cochrane**

| ID | Search |
| --- | --- |
| #1 | MeSH descriptor: [Gram-Negative Bacteria] explode all trees |
| #2 | (Gram-Negative Bacteria OR gram-negative bacillus OR gram-negative bacterium OR GNB):ti,ab,kw |
| #3 | MeSH descriptor: [Acinetobacter baumannii] explode all trees |
| #4 | (Acinetobacter baumannii OR Acinetobacter baumanii):ti,ab,kw |
| #5 | MeSH descriptor: [Pseudomonas aeruginosa] explode all trees |
| #6 | Pseudomonas aeruginosa:ti,ab,kw |
| #7 | MeSH descriptor: [Escherichia coli] explode all trees |
| #8 | (Escherichia coli OR e.coli):ti,ab,kw |
| #9 | MeSH descriptor: [Klebsiella pneumoniae] explode all trees |
| #10 | Klebsiella pneumoniae:ti,ab,kw |
| #11 | MeSH descriptor: [Staphylococcus aureus] explode all trees |
| #12 | MeSH descriptor: [Methicillin-Resistant Staphylococcus aureus] explode all trees |
| #13 | (Methicillin-Resistant Staphylococcus aureus OR Staphylococcus aureus OR MRSA):ti,ab,kw |
| #14 | MeSH descriptor: [Enterobacteriaceae] explode all trees |
| #15 | MeSH descriptor: [Carbapenem-Resistant Enterobacteriaceae] explode all trees |
| #16 | (Carbapenem-Resistant Enterobacteriaceae OR Enterobacteriaceae):ti,ab,kw |
| #17 | #1 OR #2 OR #3 OR #4 OR #5 OR #6 OR #7 OR #8 OR #9 OR #10 OR #11 OR #12 OR #13 OR #14 OR #15 OR #16 |
| #18 | MeSH descriptor: [Drug Resistance, Multiple] explode all trees |
| #19 | (Drug Resistance, Multiple OR Multiple Drug Resistance OR Resistance, Multiple Drug OR Multidrug Resistance OR Multi-Drug Resistance OR Drug Resistance, Multiple):ti,ab,kw |
| #20 | #18 OR #19 |
| #21 | #17 AND #20 |
| #22 | MeSH descriptor: [Respiratory Tract Infections] explode all trees |
| #23 | Respiratory Tract Infections:ti,ab,kw |
| #24 | MeSH descriptor: [Pneumonia] explode all trees |
| #25 | MeSH descriptor: [Bronchopneumonia] explode all trees |
| #26 | MeSH descriptor: [Healthcare-Associated Pneumonia] explode all trees |
| #27 | MeSH descriptor: [Pneumonia, Ventilator-Associated] explode all trees |
| #28 | MeSH descriptor: [Pneumonia, Bacterial] explode all trees |
| #29 | (Pneumonia OR Bronchopneumonia OR Healthcare-Associated Pneumonia OR Pleuropneumonia OR Pneumonia, Ventilator-Associated OR Pneumonia, Bacterial OR VAP OR HAP):ti,ab,kw |
| #30 | #22 OR #23 OR #24 OR #25 OR #26 OR #27 OR #28 OR #29 |
| #31 | #21 AND #30 in Trials |

**Table S4 Studies excluded in full text screening**

| **Author** | **Year** | **Title** | **Reasons for exclusion** |
| --- | --- | --- | --- |
| Wang | 2018 | Risk Factors for Carbapenem-resistant Klebsiella pneumoniae Infection and Mortality of Klebsiella pneumoniae Infection | a |
| Tseng | 2018 | Risk for subsequent infection and mortality after hospitalization among patients with multidrug-resistant gram-negative bacteria colonization or infection | a |
| Huang | 2018 | A multi-center study on the risk factors of infection caused by multi-drug resistant Acinetobacter baumannii | a |
| Djordjevic | 2018 | Risk factors for carbapenem-resistant klebsiella pneumoniae hospital infection in the intensive care unit | a |
| Tebano | 2016 | Epidemiology and risk factors of multidrug-resistant bacteria in respiratory samples after lung transplantation | a |
| Vasudevan | 2014 | A prediction tool for nosocomial multi-drug Resistant Gram-Negative Bacilli infections in critically ill patients - prospective observational study | a |
| Rabinowitz | 2012 | A hidden reservoir of methicillin-resistant Staphylococcus aureus and vancomycin-resistant Enterococcus in patients newly admitted to an acute rehabilitation hospital | a |
| Prata-Rocha | 2012 | Factors influencing survival in patients with multidrug-resistant Acinetobacter baumannii infection | a |
| Giannella | 2012 | Pneumonia treated in the internal medicine department: Focus on healthcare-associated pneumonia | a |
| Cardoso | 2012 | Additional risk factors for infection by multidrug-resistant pathogens in healthcare-associated infection: a large cohort study. | a |
| Yogeesha | 2011 | Study of imipenem resistant Pseudomonas aeruginosa and associated predisposing risk factors in a rural tertiary care hospital | a |
| Nseir | 2010 | Accuracy of American Thoracic Society/Infectious Diseases Society of America criteria in predicting infection or colonization with multidrug-resistant bacteria at intensive-care unit admission. | a |
| Montero | 2010 | Risk factors for multidrug-resistant Pseudomonas aeruginosa acquisition. Impact of antibiotic use in a double case-control study. | a |
| Mehrgan | 2010 | High prevalence of extended-spectrum beta-lactamase-producing Klebsiella pneumoniae in a tertiary care hospital in Tehran, Iran | a |
| Tacconelli | 2008 | Prediction models to identify hospitalized patients at risk of being colonized or infected with multidrug-resistant Acinetobacter baumannii calcoaceticus complex | a |
| Parker | 2008 | Ventilator-associated pneumonia caused by multidrug-resistant organisms or Pseudomonas aeruginosa: prevalence, incidence, risk factors, and outcomes. | a |
| Baran | 2008 | Risk factors for nosocomial imipenem-resistant Acinetobacter baumannii infections | a |
| Dantas | 2003 | Impact of antibiotic-resistant pathogens colonizing the respiratory secretions of patients in an extended-care area of the emergency department. | a |
| Ciginskiene | 2019 | Ventilator-Associated Pneumonia due to Drug-Resistant Acinetobacter baumannii: Risk Factors and Mortality Relation with Resistance Profiles, and Independent Predictors of In-Hospital Mortality | b |
| Moreau | 2018 | Impact of immunosuppression on incidence, aetiology and outcome of ventilator-associated lower respiratory tract infections. | b |
| Kumar | 2018 | Healthcare-Associated Pneumonia and Hospital-Acquired Pneumonia: Bacterial Aetiology, Antibiotic Resistance and Treatment Outcomes: A Study From North India | b |
| Khawcharoenporn | 2018 | Active monotherapy and combination therapy for extensively drug-resistant Pseudomonas aeruginosa pneumonia | b |
| Ju | 2018 | Subsequent Multidrug-Resistant Bacteremia Is a Risk Factor for Short-Term Mortality of Patients with Ventilator-Associated Pneumonia Caused by Acinetobacter baumannii in Intensive Care Unit: A Multicenter Experience | b |
| Jean | 2017 | Treatment outcomes of patients with non-bacteremic pneumonia caused by extensively drug-resistant Acinetobacter calcoaceticus-Acinetobacter baumannii complex isolates: Is there any benefit of adding tigecycline to aerosolized colistimethate sodium? | b |
| Khurana | 2017 | Incidence of ventilator-associated pneumonia and impact of multidrug-resistant infections on patient's outcome: Experience at an Apex Trauma Centre in North India | b |
| Jesus | 2017 | Analysis of treatment failure with standard and high dose of tigecycline in critically ill patients with multidrug-resistant bacteria | b |
| Tsioutis | 2016 | Clinical epidemiology, treatment and prognostic factors of extensively drug-resistant Acinetobacter baumannii ventilator-associated pneumonia in critically ill patients | b |
| Shojaei | 2016 | Clinical response and outcome of pneumonia due to multi-drug resistant acinetobacter baumannii in critically ill patients | b |
| Almomani | 2015 | Incidence and predictors of 14-day mortality in multidrug-resistant Acinetobacter baumannii in ventilator-associated pneumonia | b |
| Almomani | 2017 | Prognostic indicators and survival in salvage surgery for laryngeal cancer | b |
| Kollef | 2014 | Global prospective epidemiologic and surveillance study of ventilator-associated pneumonia due to Pseudomonas aeruginosa | b |
| Khawcharoenporn | 2014 | Colistin-based treatment for extensively drug-resistant Acinetobacter baumannii pneumonia | b |
| Chittawatanarat | 2014 | Microbiology, resistance patterns, and risk factors of mortality in ventilator-associated bacterial pneumonia in a Northern Thai tertiary-care university based general surgical intensive care unit | b |
| Vasudevan | 2013 | Inappropriate empirical antimicrobial therapy for multidrug-resistant organisms in critically ill patients with pneumonia is not an independent risk factor for mortality: Results of a prospective observational study of 758 patients | b |
| Maruyama | 2013 | A new strategy for healthcare-associated pneumonia: a 2-year prospective multicenter cohort study using risk factors for multidrug-resistant pathogens to select initial empiric therapy | b |
| Aliberti | 2013 | Multidrug-resistant pathogens in hospitalised patients coming from the community with pneumonia: a European perspective | b |
| Aliberti | 2012 | Stratifying risk factors for multidrug-resistant pathogens in hospitalized patients coming from the community with pneumonia | b |
| Ye | 2011 | The clinical implication and prognostic predictors of tigecycline treatment for pneumonia involving multidrug-resistant Acinetobacter baumannii. | b |
| Set | 2011 | Bacteriology profile among patients with ventilator-associated pneumonia from a medical intensive care unit at a tertiary care center in Mumbai | b |
| Tsakiridou | 2018 | Pre-intensive care unit intubation and subsequent delayed intensive care unit admission is independently associated with increased occurrence of ventilator-associated pneumonia | c |
| Patro | 2018 | Bacteriological profile of ventilator-associated pneumonia in a tertiary care hospital | c |
| Llitjos | 2018 | Prior antimicrobial therapy duration influences causative pathogens identification in ventilator-associated pneumonia | c |
| Ekren | 2018 | Evaluation of the 2016 Infectious Diseases Society of America/American Thoracic Society guideline criteria for risk of multidrug-resistant pathogens in patients with hospital-acquired and ventilator-associated pneumonia in the ICU. | c |
| Abd-Elmonsef | 2018 | Mechanical ventilator as a major cause of infection and drug resistance in intensive care unit | c |
| Arvanitis | 2014 | The impact of antimicrobial resistance and aging in VAP outcomes: experience from a large tertiary care center | c |
| Huang | 2013 | Sources of multidrug-resistant Acinetobacter baumannii and its role in respiratory tract colonization and nosocomial pneumonia in intensive care unit patients | c |
| Lodise | 2007 | Clinical prediction tool to identify patients with Pseudomonas aeruginosa respiratory tract infections at greatest risk for multidrug resistance | c |
| Mukhopadhyay | 2013 | Role of mechanical ventilation & development of multidrug resistant organisms in hospital acquired pneumonia | c |
| Husni | 1999 | Risk factors for an outbreak of multi-drug-resistant Acinetobacter nosocomial pneumonia among intubated patients. | c |
| Gao | 2019 | Molecular epidemiology and risk factors of ventilator-associated pneumonia infection caused by carbapenem-resistant enterobacteriaceae | d |
| Bassetti | 2018 | Risk stratification and treatment of ICU-acquired pneumonia caused by multidrug- resistant/extensively drug-resistant/pandrug-resistant bacteria | d |
| Li | 2018 | Clinical analysis of bacterial pneumonia with multidrug-resistant bacterial infection in elderly patients | d |
| Trinh | 2017 | Multidrug-resistant Pseudomonas aeruginosa lower respiratory tract infections in the intensive care unit: Prevalence and risk factors | d |
| Li | 2017 | Pneumonia caused by extensive drug-resistant Acinetobacter baumannii among hospitalized patients: genetic relationships, risk factors and mortality | d |
| Terraneo | 2017 | Severity of COPD is associated with multidrug resistant organisms in hospitalized patients with community-acquired pneumonia | d |
| Lee | 2017 | Proposed prediction tool for multidrug-resistant pathogens in patients admitted with hemodialysis-associated pneumonia | d |
| Di Pasquale | 2017 | A worldwide evaluation of multi-drug resistant organisms in nursing home acquired pneumonia | d |
| Adukauskiene | 2017 | Ventilator-associated pneumonia: MDR gram-negative bacteria and predictors of mortality. | d |
| Sarkar | 2016 | Ventilator associated Pneumonia a challenge in intensive care unit acquired infection | d |
| Ferrer | 2014 | ICU acquired pneumonia due to pseudomonas aeruginosa with and without multidrug resistance | d |
| De Rosa | 2014 | Risks and prognostic factors for ICU-acquired pneumonia due to pseudomonas aeruginosa with and without multidrug resistance | d |
| Grgurich | 2012 | Management and prevention of ventilator-associated pneumonia caused by multidrug-resistant pathogens | d |
| Mentzelopoulos | 2007 | Prolonged use of carbapenems and colistin predisposes to ventilator-associated pneumonia by pandrug-resistant Pseudomonas aeruginosa | e |
| Werarak | 2010 | Hospital-acquired pneumonia and ventilator-associated pneumonia in adults at Siriraj Hospital: etiology, clinical outcomes, and impact of antimicrobial resistance | f |

a: Wrong population; b: No interesting outcomes; c: No multivariate analysis results; d: Wrong type studies; e: MDR events less than 10; f: Full-text unavailable.

**Table S5 Additional risk factors that had a statistically significant association with the acquisition of MDR bacteria**

| **Risk factors** | **No. of included studies** | **No. of included MDROs** | **Synthesized results** |
| --- | --- | --- | --- |
| **Antibiotic treatment** | | | |
| Prophylactic antibiotic days | 1 | 135 | OR 23.1, 95%CI, 16.7 to 28 |
| Cefepime | 1 | 97 | OR 2.87, 95%CI, 1.11 to 7.37 |
| More than two antibiotic classes before VAP | 1 | 52 | OR 3.93, 95%CI, 1.26 to 12.23 |
| **Prior infection/colonization** | | | |
| Candida spp. airway colonization | 1 | 90 | OR 1.79, 95%CI, 1.05 to 3.05 |
| **Hospitalization** | | | |
| Current hospitalization ≥ 2 days | 1 | 154 | OR 1.98, 95%CI, 1.12 to 3.49 |
| Time elapsed between ICU admission and VAP suspicion | 1 | 90 | OR 1.04, 95%CI, 1.02 to 1.06 |
| **Hospital interventions** | | | |
| Prolonged duration of intubation ≥ 72 h | 1 | 57 | OR 8.22, 95%CI, 4.28 to 15.82 |

MDROs: Multi-drug resistant organisms; VAP: Ventilator-associated pneumonia; ICU: Intensive care unit.

**Table S6: Results of sensitivity analysis** **using the fixed effects models**

| **Risk factors** | **No. of included studies** | **Heterogeneity** | **Synthesized results** |
| --- | --- | --- | --- |
| Prior antibiotic treatment | 12 | I^2^=20.8%, P=0.239 | OR 2.47, 95%CI, 1.92 to 3.18 |
| Inappropriate antibiotic therapy | 2 | I^2^=41.1%, P=0.193 | OR 16.34, 95%CI, 11.54 to 23.12 |
| Chronic lung disease | 5 | I^2^=0.0%, P=0.611 | OR 2.19, 95%CI, 1.51 to 3.19 |
| Chronic liver disease | 2 | I^2^=0.0%, P=0.403 | OR 3.41, 95%CI, 1.55 to 7.51 |
| Cardiac disease | 2 | I^2^=57.9%, P=0.123 | OR 0.59, 95%CI, 0.32 to 1.07 |
| Cerebral disease | 3 | I^2^=73.2%, P=0.024 | OR 2.46, 95%CI, 1.68 to 3.59 |
| Renal replacement therapy | 3 | I^2^=0%, P=0.559 | OR 0.78, 95%CI, 0.41 to 1.48 |
| Prior MDR infection | 3 | I^2^=60.8%, P=0.078 | OR 3.34, 95%CI, 1.97 to 5.66 |
| Prior PA infection | 2 | I^2^=0.0%, P=0.506 | OR 10.29, 95%CI, 5.03 to 21.07 |
| Recent hospitalization | 4 | I^2^=10.6%, P=0.340 | OR 2.46, 95%CI, 1.51 to 4.01 |
| Hospitalization stay | 4 | I^2^=76.3%, P=0.005 | OR 1.02, 95%CI, 1.01 to 1.03 |
| Health care exposure prior to admission | 3 | I^2^=0.0%, P=0.618 | OR 3.10, 95%CI, 1.94 to 4.97 |
| Endotracheal intubation | 3 | I^2^=91.3%, P=0.000 | OR 4.32, 95%CI, 2.67 to 7.00 |
| Mechanical ventilation | 3 | I^2^=65.1%, P=0.057 | OR 6.75, 95%CI, 3.59 to 12.67 |
| Tube feeding | 2 | I^2^=0.0%, P=0.659 | OR 2.95, 95%CI, 1.12 to 7.80 |
| Disease severity scores | 2 | I^2^=11.2%, P=0.289 | OR 2.26, 95%CI, 1.44 to 3.54 |
| HAP or VAP | 3 | I^2^=29.1%, P=0.244 | OR 1.90, 95%CI, 0.84 to 4.30 |

HAP Hospital-acquired pneumonia; VAP Ventilator-associated pneumonia.

**Table S7: Results of sensitivity analysis using the** **HKSJ** **random effects model**

| **Risk factors** | **No. of included studies** | **Heterogeneity** | **Synthesized results** |
| --- | --- | --- | --- |
| Prior antibiotic treatment | 12 | I^2^=20.8%, P=0.239 | OR 2.61, 95%CI, 1.88 to 3.62 |
| Inappropriate antibiotic therapy | 2 | I^2^=41.1%, P=0.193 | OR 14.91, 95%CI, 0.38 to 582.00 |
| Chronic lung disease | 5 | I^2^=0.0%, P=0.611 | OR 2.22, 95%CI, 1.44 to 3.41 |
| Chronic liver disease | 2 | I^2^=0.0%, P=0.403 | OR 3.47, 95%CI, 0.04 to 268.42 |
| Cardiac disease | 2 | I^2^=57.9%, P=0.123 | OR 0.67, 95%CI, 0.00 to 472.85 |
| Cerebral disease | 3 | I^2^=73.2%, P=0.024 | OR 3.08, 95%CI, 0.38 to 24.96 |
| Renal replacement therapy | 3 | I^2^=0%, P=0.559 | OR 0.80, 95%CI, 0.25 to 2.56 |
| Prior MDR infection | 3 | I^2^=60.8%, P=0.078 | OR 3.77, 95%CI, 0.59 to 23.88 |
| Prior PA infection | 2 | I^2^=0.0%, P=0.506 | OR 10.24, 95%CI, 0.46 to 229.20 |
| Recent hospitalization | 4 | I^2^=10.6%, P=0.340 | OR 2.50, 95%CI, 1.05 to 5.91 |
| Hospitalization stay | 4 | I^2^=76.3%, P=0.005 | OR 1.03, 95%CI, 1.00 to 1.07 |
| Health care exposure prior to admission | 3 | I^2^=0.0%, P=0.618 | OR 3.14, 95%CI, 1.53 to 6.44 |
| Endotracheal intubation | 3 | I^2^=91.3%, P=0.000 | OR 6.52, 95%CI, 0.18 to 234.07 |
| Mechanical ventilation | 3 | I^2^=65.1%, P=0.057 | OR 7.79, 95%CI, 0.79 to 76.57 |
| Tube feeding | 2 | I^2^=0.0%, P=0.659 | OR 2.95, 95%CI, 0.18 to 47.69 |
| Disease severity scores | 2 | I^2^=11.2%, P=0.289 | OR 2.34, 95%CI, 0.09 to 62.62 |
| HAP or VAP | 3 | I^2^=29.1%, P=0.244 | OR 1.66, 95%CI, 0.12 to 23.50 |

HKSJ Hartung-Knapp-Sidik-Jonkman; HAP Hospital-acquired pneumonia; VAP Ventilator-associated pneumonia
